# Usefulness of carotid ultrasonography in the diagnosis of coronary artery disease in patients undergoing exercise echocardiography

**DOI:** 10.1186/s12947-018-0143-x

**Published:** 2018-10-09

**Authors:** Raúl Franco-Gutiérrez, Alberto José Pérez-Pérez, Virginia Franco-Gutiérrez, Ana María Testa-Fernández, Rafael Carlos Vidal-Pérez, Manuel Lorenzo López-Reboiro, Víctor Manuel Puebla-Rojo, Melisa Santás-Álvarez, María Generosa Crespo-Leiro, Carlos González-Juanatey

**Affiliations:** 1 Department of Cardiology, Hospital Universitario Lucus Augusti (HULA), Avenida doctor Ulises Romero n° 1, 27003 Lugo, Spain; 2 Department of Otolaryngology, Hospital Universitario Marqués de Valdecilla, Avenida Valdecilla n° 25, Santander, 39008 Spain; 3 Department of Internal Medicine, Hospital Universitario Lucus Augusti (HULA), Avenida doctor Ulises Romero n° 1, Lugo, 27003 Spain; 4 Department of Cardiology, Complejo Hospitalario Universitario A Coruña (CHUAC), As Xubias de Arriba n° 84, A Coruña, 15006 Spain; 5 Intitituto de Investigación Biomédica A Coruña (INIBIC), Xubias de Arriba n° 84, A Coruña, 15006 Spain; 6 Universidad de La Coruña (UDC), Calle de la Maestranza n° 9, A Coruña, 15001 Spain

**Keywords:** Stress echocardiography, Exercise test, Carotid artery disease, Coronary artery disease, Area under curve

## Abstract

**Background:**

Relationship between carotid and coronary artery disease (CAD) in patients undergoing invasive and non-invasive test is unclear. The aim of the study is to evaluate whether carotid disease is associated with CAD in patients submitted to exercise echocardiography (EE) and if it improves the EE ability to predict CAD.

**Methods:**

We retrospectively studied 156 subjects without previous vascular disease who underwent EE, carotid ultrasonography and coronary angiography between 2002 and 2013. Positive EE was defined as exercise induced wall motion abnormalities, carotid disease according to Manheim and American Society of Echocardiography Consensus and significant CAD as stenosis ≥50%.

**Results:**

Eighty-nine (57.1%) subjects had significant CAD. Factors associated with CAD in multivariate analysis were fasting plasma glucose (odds ratio [OR] 1.02, *p* = 0.031), pre-test probability of CAD > 65% (OR 3.71, *p* < 0.001), positive EE (OR 10.51, *p* < 0.001) and carotid plaque (CP) presence (OR 2.95, *p* = 0.013). There was neither statistical significant difference in area under the curve after addition of CP to EE results (0.77 versus 0.81, *p* = 0.525) nor sensitivity, specificity, predictive values or efficiency. CP presence reclassified as very high-risk according to Systematic COronary Risk Evaluation 13 patients (34.2%) with negative EE and 22 (33.3%) without CAD.

**Conclusion:**

CP is associated with CAD in patients undergoing EE, however its addition to EE does not improve CAD prediction, probably due to insufficient statistical power. CP reclassified one third of patients to very high-risk category despite negative EE or CAD absence, these subjects benefit from aggressive primary prevention interventions.

## Background

Ischaemic heart disease is a major problem due to its prevalence, health cost and mortality [1–3]. Stress echocardiography is a well-validated tool for diagnosis and risk stratification in patients with new onset chest pain, but it has some limitations that can impair its diagnostic capacity such as the dependence of pre-test probabilities (PTP) of coronary artery disease (CAD), the need to achieve submaximal heart rate, the presence of suboptimal echocardiographic windows, the inability to detect non limiting flow coronary stenosis or pathologies that can produce wall motion abnormalities during exercise [2–4].

Carotid disease, defined as increased carotid intima-media thickness (CIMT) or the presence of atherosclerotic plaques (CP), has been associated with myocardial infarction, stroke and death [5–7]. Post-mortem studies have also demonstrated a correlation between carotid and CAD [8]. These findings encouraged investigators to evaluate the possibility of using carotid disease in the diagnosis of CAD of patients undergoing invasive and non-invasive tests, however the studies published so far have shown inconsistent results [9–19]. In that sense a meta-analysis of 34 studies focused on the relation of CIMT with coronary atherosclerosis, 30 showed a positive but modest relationship with correlation positive coefficients between 0.12 and 0.51 with only one study being above 0.5 and some studies showed no relationship at all [19].

Our group has broad experience in the ultrasonographic assessment of carotid arteries, having demonstrated its usefulness as a marker of subclinical atherosclerosis in subjects with autoimmune diseases [20]. The studies mentioned before [5–8], along with our findings, led to the systematic use of carotid ultrasound in subjects with suspected CAD undergoing exercise echocardiography (EE) at our cardiovascular imaging laboratory since 2002. This approach has been endorsed by the European Society of Cardiology (ESC) stable CAD guidelines as a IIa level C recommendation [2].

A clinical study was designed to evaluate if carotid disease is associated with significant CAD in patients with suspected ischaemic heart disease undergoing treadmill exercise stress echocardiography at our institution and if it improves the EE ability to predict significant CAD.

## Methods

### Study population

Between Jan. 1st 2002 and Dec. 31st 2013 4024 consecutive Caucasian subjects older than 18 years with suspected CAD underwent EE and carotid ultrasonography at our institution. Of them, 390 patients (9.7%) were also submitted to a coronary angiography. 234 patients (60%) were excluded: 29 (7.4%) due to prior stroke, transient ischaemic attack or peripheral artery disease and 205 due to prior CAD (52.6%) defined as previous myocardial infarction [21], coronary revascularization or angiographic documentation of any coronary stenosis ≥50%. All patients signed informed consent before testing. The study was approved by the Regional Ethics Committee.

Demographic, clinical, baseline echocardiography, carotid ultrasonography and stress testing data were collected. PTP of CAD and Systematic COronary Risk Evaluation (SCORE) were assessed according to current ESC guidelines [1, 2].

### Treadmill exercise stress echocardiography

Treadmill exercise was the stress modality chosen using a Philips Sonos 5500 ultrasound machine between 2002 and 2005 and a Philips iE33 after 2005 (Philips Medical Systems).

Heart rate, blood pressure and 12-lead electrocardiogram were obtained at baseline and at each exercise stage. EE was finished in case of physical exhaustion, disabling chest pain, significant arrhythmia and severe hypertensive or hypotensive response. Apical long-axis, apical 4- and 2-chamber and parasternal long- and short-axis views were obtained at rest, peak and immediately after exercise. Echocardiographic analysis was performed using a 17-segment model of the left ventricle to evaluate regional wall motion. Each segment was graded on a 4-point scale depending on its motion. Wall motion score index was calculated as the sum of the scores divided by the number of segments at rest and at peak exercise.

Ischaemic electrocardiographic abnormalities were defined as development of ST-segment deviation 80 msec after J point ≥1 mm. Echocardiographic ischaemia was defined as exercise induced new or worsening wall motion abnormalities, except worsening from akinesia to dyskinesia and isolated hypokinesia of the inferobasal segment. Extensive ischemia was defined as ischaemia involving ≥3 myocardial segments and multivessel ischemia as ischemia involving ≥2 different coronary territories [4].

### Carotid ultrasonography

Carotid scans were performed immediately after stress testing in the same EE ultrasound equipment using a high-resolution, B-mode ultrasound system with a linear array (3–11 MHz) transducer. Measurement of the CIMT and CP definition were done following the ARIC protocol study [5] and expert consensus [22–25]. Semi-automated edge detection software was used (QLAB; Philips 110 Medical Systems, Andover, MA, USA).

Age- and sex-specific CIMT percentile values were obtained from previously published data in our country [26].

Both EE and carotid ultrasonography stored images were analysed by two imaging expert cardiologists blinded to angiography results. In case of disagreement a third expert was consulted.

### Coronary angiography

The physician in charge of the patient carried out a coronary angiography considering the results of the EE and other conditions such as persistence of symptoms despite optimal medical treatment, patients’ preferences and/or other clinical criteria. Coronary angiography was performed using standard technique. Significant angiographic disease was defined as stenosis ≥50% by visual assessment in any major epicardial arteries or in their branches.

Coronary angiography analysis was similar to ultrasonography.

### Statistical analysis

Categorical variables were reported as percentages and comparison between groups were based on chi-square or Fisher’s exact tests. Continuous variables were reported as mean (standard deviation) or median [interquartile range] when their distribution departed from normal and differences were assessed via the unpaired *t* test or the Mann-Whitney U test where appropriate. Binary and continuous quantitative variables were compared using logistic binary regression. To create predictive models for the presence of significant CAD, backward stepwise binary logistic regression was used with an entry set at 0.2 significance level and a retention set of 0.1. A *p* value of < 0.05 was considered statistically significant. ! *DT V2009.06.26®* macro for SPSS Statistics (Autonomous University of Barcelona) and IBM SPSS Statistics for Windows, Version 20.0. (Armonk, NY) was used to calculate sensitivity, specificity, positive (PPV) and negative predictive values (NPV), positive (PLR) and negative likelihood ratios (NLR) and efficiency of EE alone and combined with carotid ultrasonography. Area under the curve (AUC) was calculated by means of a receiver operating characteristic curve analysis; comparison between AUC was done by the DeLong method.

## Results

One hundred fifthy six patients were enrolled in the study. Mean age was 66.1 ± 10.4 years and 102 (65.4%) were men. There were no major complications during or after the tests.

Baseline characteristics are summarized in Table 1.

**Table 1:** Baseline characteristics of patients

	Non-prior vascular disease (*n* = 156)
Age (years)	66.1 (10.4)
Male sex (%)	102 (65.4%)
Body mass index (Kg/m^2^)	28.7 (4.0)
Hypertension	93 (59.6%)
Hypercholesterolemia	91 (58.3%)
DM	41 (26.3%)
Smoking habit	68 (43.6%)
Family history of premature CAD	22 (14.1%)
SCORE	
Low	10 (6.4%)
Moderate	52 (33.3%)
High	47 (30.1%)
Very high	45 (28.8%)
Chest pain	149 (95.5%)
Typical	82 (55.0%)
Atypical	65 (43.6%)
Non-anginal	2 (1.3%)
FPG levels (mg/dL)	114.3 (33.5)
GFR (ml//min/1.73 m^2^)	78.3 (24.0)
Total Cholesterol levels (mg/dL)	189.2 (44.7)
Low-density lipoprotein levels (mg/dL)	114.4 (38.5)
High-density lipoprotein levels (mg/dL)	44.1 (11.7)
Triglyceride levels (mg/dL)	159.1 (94.1)
Drugs prior EE	
Beta-blockers	36 (23.1%)
Calcium channel blockers	40 (25.6%)
Nitrates	23 (14.7%)
Statins	68 (43.6%)
Antiplatelet drugs	51 (32.7%)
EE data	
Systolic BP (mmHg)	
Rest	141.5 (20.3)
Peak	184.9 (29.3)
Heart rate (beats/min)	
Rest	69.9 (13.1)
Peak	131.6 (18.6)
Rate-pressure (× 10^3^ mmHg beats/min)	
Rest	9.9 (2.5)
Peak	24.4 (5.6)
Exercise time (min)	6.9 (2.7)
Positive EE	93 (59.6%)
Negative EE	38 (24.4%)
Failure to achieve submaximal predicted heart rate	25 (16.0%)
Metabolic equivalents	7.5 (2.6)
Left ventricular ejection fraction (%)	
Rest	62.5 (7.1)
Peak	64.3 (12.4)
Resting wall motion abnormality	21 (13.6%)
Wall motion score index	
Rest	1.04 (0.17)
Peak	1.22 (0.28)
Carotid ultrasound data	
Mean CIMT (mm)	0.88 (0.19)
Mean CIMT percentile Spanish population	
≤ 25^th^	18 (11.5%)
25th - 75^th^	40 (25.6%)
≥ 75^th^	98 (62.8%)
CP	95 (60.9%)
Calcified CP	47 (30.5%)

*BP* Blood pressure, *CAD* coronary artery disease, *CIMT* carotid intima-media thickness, *CP* carotid plaque, *DM* diabetes mellitus, *EE* exercise echocardiography, *FPG* fasting plasma glucose, *GFR* glomerular filtration rate, *SCORE* European Systematic COronary Risk Evaluation

### Prediction of CAD

Mean time between non-invasive tests and coronary angiography was 4.2 (3.2) months. Of the 156 patients 89 (57.1%) had significant CAD. This subgroup was older (*p* = 0.045), with male predominance (*p* = 0.011), had more frequently diabetes mellitus (DM), smoking habit (*p* = 0.023) and higher levels of fasting plasma glucose (FPG) (*p* = 0.003). Higher SCORE, PTP of CAD as well as positive EE and CP presence (all of them *p* < 0.001) were also significantly more frequent in patients with CAD.

In multivariate analysis FPG (*p* = 0.031), PTP > 65% (*p* < 0.001), positive EE (*p* < 0.001) and CP (*p* = 0.013) were predictors of significant CAD.

Comparisons of subgroups with and without significant CAD and multivariate analysis are represented in Tables 2 and 3 respectively.

**Table 2:** Clinical, demographic, exercise and carotid ultrasound data in the subgroup of patients with and without CAD

	CAD ≥ 50% (*n* = 89)	CAD < 50% (*n* = 67)	*p* value
Age	67.6 (9.2)	64.1 (11.6)	0.045
Male sex	66 (74.2%)	36 (53.7%)	0.011
Body mass index	29.1 (4.2)	28.1 (3.7)	0.134
Hypertension	55 (61.8%)	38 (56.7%)	0.621
Hypercholesterolemia	56 (62.9%)	35 (52.2%)	0.193
DM	31 (34.8%)	10 (14.9%)	0.006
Smoking habit	46 (51.7%)	18 (32.8%)	0.023
Family history of early CAD	14 (15.7%)	8 (11.9%)	0.643
FPG levels	120.7 (38.7)	105.7 (22.4)	0.03
Total Cholesterol levels	192.0 (47.5)	185.5 (40.8)	0.379
Low-density lipoprotein levels	117.2 (40.7)	110.7 (36.1)	0.308
High-density lipoprotein levels	43.0 (11.3)	45.6 (12.1)	0.168
Triglyceride levels	160.6 (91.1)	157.2 (98.7)	0.824
GFR	75.6 (23.2)	81.1 (24.7)	0.105
SCORE			< 0.001
Low	1 (1.1%)	9 (13.6%)	
Moderate	24 (27.3%)	28 (42.4%)	
High	28 (31.8%)	19 (28.8%)	
Very high	35 (39.8%)	10 (15.2%)	
PTP of CAD			< 0.001
< 15%	0 (0%)	3 (4.5%)	
15–65%	31 (34.8%)	42 (62.7%)	
65–85%	55 (61.8%)	22 (32.8%)	
> 85%	3 (3.4%)	0 (0%)	
Positive EE	73 (82.0%)	20 (29.9%)	< 0.001
Mean CIMT (mm)	0.88 (0.21)	0.89 (0.18)	0.926
CIMT > 0.9 mm	38 (42.7%)	31 (46.3%)	0.745
CIMT > 75^th^ percentile	52 (58.4%)	46 (68.7%)	0.242
CP	66 (74.2%)	29 (43.3%)	< 0.001
Calcified CP	32 (36.0%)	15 (22.4%)	0.079

*PTP* Pre-test probability. Rest of abbreviations as in Table 1

**Table 3:** Multivariate significant CAD analysis

Variable	B	*p* value	OR	95% CI	
				Lower	Higher
Constant	−4.83	< 0.001	0.01		
Smoking habit	0.84	0.057	2.31	0.98	5.46
FPG	0.02	0.031	1.02	1.00	1.04
PTP of CAD > 65%	1.31	0.003	3.71	1.57	8.79
Positive EE	2.35	< 0.001	10.51	4.38	25.20
CP	1.08	0.013	2.95	1.25	6.93

*CI* confidence interval, *OR* odds ratio. Rest of abbreviations as in Tables 1 and 2

Regarding the subgroup of 21 (13.6%) subjects with resting wall motion abnormalities 4 (19%) had global left ventricular hypokinesia. Of the 21 patients 17 (81.0%) developed worsening wall motion abnormalities during EE and all of them showed significant CAD in the angiography, 2 (9.5%) were defined as negative EE and did not have significant CAD and 2 (9.5%) could not achieve submaximal predicted heart rate, both without significant CAD in the angiography.

### AUC, sensitivity, specificity, predictive values, PLR and NLR and efficiency

**Fig. 1:**
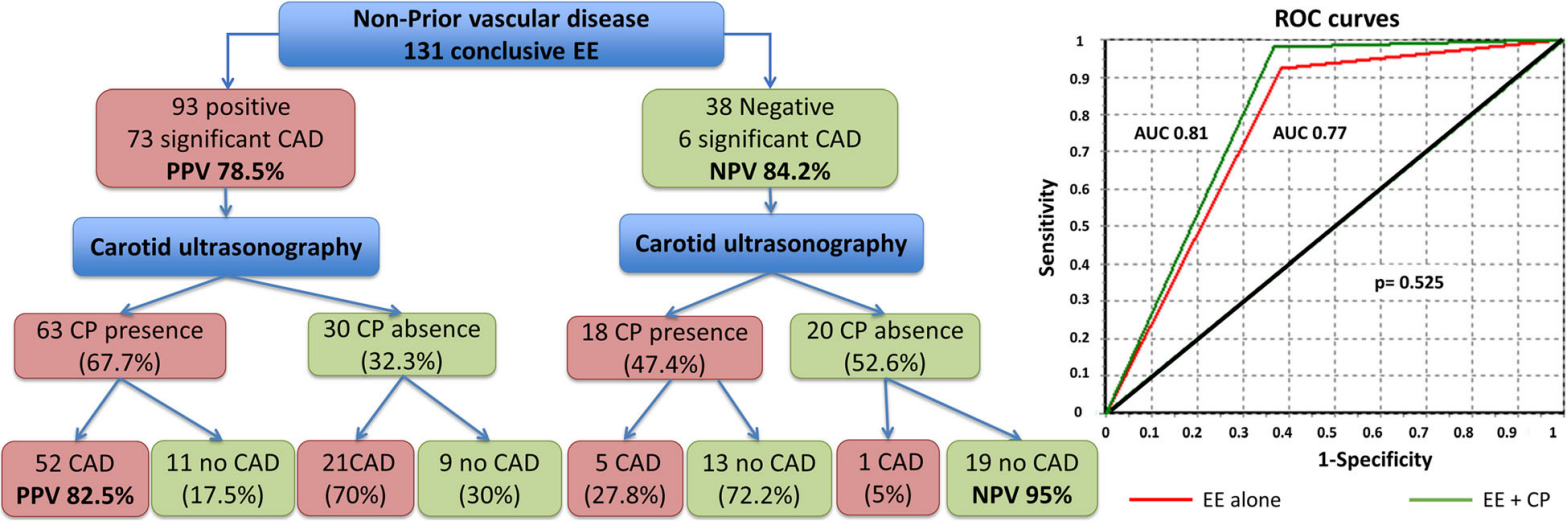
Relationship between EE and CP and ROC curve representation. ROC: Receiver Operating Characteristic. Rest of abbreviation as in Tables 1, 2, 3 and 4

AUC of EE alone was 0.77 (95% confidence interval [CI] 0.68–0.86), whereas AUC combining CP findings was 0.81 (95%CI 0.70–0.92) (*p* = 0.525). Results are summarized in Fig. 1 and Table 4.

**Table 4:** Sensitivity, specificity, predictive values, AUC and likelihood ratios for CAD diagnosis

Conclusive EE (*N* = 131)								
	Sensitivity (95% CI)	Specificity (95%CI)	PPV (95% CI)	NPV (95% CI)	Efficiency (95% CI)	AUC (95% CI)	PLR	NLR
EE	92.4% (84.4–96.5)	61.5% (48.0–73.5)	78.5% (69.1–85.6)	84.2% (69.6–92.6)	80.2% (72.5–86.1)	0.77 (0.68–0.86)	2.40	0.12
EE + CP	98.1% (90.1–99.7)	63.3% (45.5–78.1)	82.5% (71.4–90.0)	95.0% (76.4–99.1)	85.5% (76.4–91.5)	0.81 (0.70–0.92)	2.68	0.03

*AUC* area under the curve, *NLR* negative likelihood ratio, *NPV* negative predictive value, *PLR* positive likelihood ratio, *PPV* positive predictive value. Rest of abbreviations as in Tables 1, 2 and 3

Sensitivity, specificity, predictive values, PLR and NLR and efficiency of EE alone and EE combined with CP are also summarized in Table 4. Table 5 shows predictive values according to established intermediate PTP.

**Table 5:** Predictive values for significant CAD prediction depending on its prevalence

Prevalence of CAD	PPV (95%CI)	NPV (95%CI)
EE alone		
15%	29.8% (23.0–37.6)	97.9% (95.0–99.0)
65%	81.7% (75.9–86.4)	81.4% (66.3–90.7)
85%	93.2% (90.6–95.1)	58.8% (39.1–76.1)
EE + CP		
15%	32.1% (22.8–43.1)	99.5% (96.4–99.9)
65%	83.3% (75.6–88.9)	94.8% (71.8–99.2)
85%	93.8% (90.4–96.0)	85.6% (45.5–97.7)

Abbreviations as in Table 4

### SCORE reclassification according to carotid ultrasound

According to European guidelines on cardiovascular disease prevention [1] 10 subjects (6.4%) had low-risk at the time of EE, 52 (33.3%) had moderate-risk, 47 (30.1%) had high-risk, 45 (28.8%) had very high-risk and 2 patients (1.3%) could not be classified. When carotid ultrasonography findings were applied 59 patients (37.8%) were reclassified as very high-risk according to CP presence. Focusing in the 62 patients with low or moderate SCORE risk, 28 (45.2%) had CP.

Of the 38 patients with negative EE 5 subjects (13.2%), 16 (42.1%), 10 (26.3%) and 7 (18.4%) had low, moderate, high and very high-risk respectively. Considering CP presence 13 patients (34.2%) were reclassified as very high-risk. Regarding the 21 patients with low or moderate SCORE risk and negative EE, 7 (33.3%) had CP being thereby considered as very high-risk.

Finally, of the 67 patients without CAD, 9 subjects (13.4%) had low-risk, 28 (41.8%) had moderate-risk, 19 (28.4%) had high-risk, 10 (14.9%) had very high-risk and 1 (1.5%) could not be classified. Considering CP results, 22 patients (33.3%) were classified as very high-risk despite normal angiography. Of the 37 patients without significant CAD initially classified as low or moderate SCORE risk 12 (32.4%) presented CP.

## Discussion

This study correlates carotid disease with CAD in a real life cohort of patients without prior vascular disease undergoing EE. However, its addition to stress test does not improve CAD prediction by angiography. It is necessary to highlight the fact that nearly one third of patients with negative EE and without CAD are reclassified to high-risk group according to carotid ultrasonography findings.

Akosah et al. [13] found an association between carotid (CP or maximal CIMT ≥1 mm) and CAD in 236 patients referred for elective coronary angiography with a high NPV in case of both negative tests. However, only 162 (68.6%) subjects had stress test performed (the type was not described in their study) with a low PPV (36%) and also 95%CI were not reported. Kanwar et al. [14] reported a study on 50 symptomatic patients without prior CAD who underwent coronary angiography after stress testing. CP, especially those with heterogeneous composition, irregular surface or calcification, was a predictor of significant CAD showing a NPV of 100% in patients with negative/equivocal stress test and CP absence. In contrast to our study, 28% were non-Caucasians and they used different modalities of stress imaging test with a high incidence (64%) of equivocal results. Coskun et al. [15] identified hypertension and CIMT ≥1 mm as predictors of significant CAD in patients without previous CAD or stroke, scheduled for coronary angiography after a positive stress test. Similarly to Akosah et al. [13], the PPV of the stress test was lower compared to our results (61%). Finally, Ahmadvazir et al. [16] identified PTP, positive stress test and presence of CP as predictors of significant CAD in 591 patients with suspected CAD undergoing stress echocardiography. As in previous studies, the NPV combining stress test and carotid ultrasonography was high (80%) and, in agreement with our findings, nearly one third of the patients were reclassified for risk score according to CP results. However, only 35% of their patients were Caucasian, exercise as stress method was only used in 62% and only 83 (14%) underwent coronary angiography and, similar to the other studies [13–15], CI or comparison between AUC were not reported. In contrast with previous results, Sachpekidis [17] did not find any statistical association between carotid and CAD (defined as positive dobutamine stress test) in 130 patients, 43% of them with previous CAD. However, the study population was small with only 38.5% yielding positive results, prior CAD could have hampered its findings and there was no comparison with angiography.

Atherosclerosis is a systemic disease and it is likely that patients with carotid disease also have CAD. This fact, as previously mentioned, was demonstrated in post-mortem studies [8] and in Bots’ meta-analysis [19]. The highly variability of the association, with a correlation range between − 0.04 - 0.51 in the aforementioned meta-analysis, could be due to methodological differences in carotid ultrasound assessment and/or variability in atherosclerosis development between the vascular territories [19]. According to European and American guidelines on the management of stable CAD [2, 3] PTP of CAD must be established and then a non-invasive test must be performed for diagnostic or prognostic purposes depending on the degree of PTP. Both agree that a history of cerebrovascular or peripheral artery disease increases the likelihood of CAD [2, 3].

In our study most of the patients (96.2%) had intermediate PTP and, most importantly, none of them had previous vascular or CAD. Predictors positively associated with significant CAD were positive EE (OR = 10.51), PTP > 65% (OR = 3.71), CP (OR = 2.95) and FPG levels (OR = 1.02). It is interesting to mention that other important risk factors associated with CAD such as hypertension, hypercholesterolemia, cholesterol levels or smoking habit [1–3] were not significantly associated with CAD in our study, this fact can be explained due to insufficient statistical power and due to treatment effect, for example 42 patients (47.2%) with significant CAD were taking statins at the time of EE performance while only 26 (38.8%) of subjects without CAD were taking them, also 56 (62.9%) subjects with significant CAD where on antihypertensive drugs compared to only 35 (52.2%) of patients without CAD. FPG not DM was associated with CAD, the reason may be because the development of macrovascular disease occurs with insulin resistance, prior to DM diagnosis [27]; high or very high-risk SCORE was not also associated with CAD, probably because it is not designed to estimate it, just the risk of a fatal atherosclerotic event [1]. Although CP is the third in order in multivariable analysis after positive EE and PTP of CAD > 65%, it increases by nearly 3 the likelihood of having significant CAD so carotid ultrasound could be useful in case of intermediate PTP, where diagnosis must be confirmed, or in equivocal EE. Moreover, and similar to Ahmadvazir et al. [16], CP presence reclassified around one third of patients to a high-risk category despite a negative EE or a normal coronary angiography. This is a very remarkable finding because these subjects benefit from aggressive primary preventive therapies [1] and, although ESC guidelines on cardiovascular disease prevention in clinical practice establish atherosclerotic plaque detection by carotid artery scanning in cardiovascular risk assessment as a IIb class level of evidence B recommendation [1], considering previously mentioned studies [7, 16] it might be changed to a IIa recommendation. Finally, although CP is associated with significant CAD its addition to EE did not improve AUC (*p* = 0.525), predictive values, efficiency and likelihood ratios due to CI overlap. These facts can be explained by insufficient statistical power, however it is important to mention the markedly but statistically non-significant increase in both NPV, especially in the moderate and high PTP of CAD groups, and in the NLR. These findings, although non-significant, are consistent to Kanwar et al. [14] and Ahmadvazir et al. [16] studies where CI were not reported. In this sense we considered our study only as hypothesis generating and increasing sample could corroborate it. Although there is a study addressing the utility of carotid ultrasonography for selecting patients who do not require coronary angiography before heart valve surgery [28], in our study 25.8% of patients with significant CAD did not have CP and 43.3% of patients without significant CAD have CP in the carotid ultrasonography. For that reason we consider non-invasive stress test as the first line test in symptomatic patients with intermediate PTP and carotid ultrasonography as an additional tool for decision making. Unlike Kanwar et al. [14] we did not specifically analysed CP morphology, nevertheless we did not find significant association between calcified CP and significant CAD, this fact can be related to insufficient sample size.

Our study has some limitations. First of all, it is a retrospective single institution study with a low recruitment rate and therefore it is hampered by the use of different equipments and methods of image storage. One alternative could be a multicentre prospective study. Secondly, not all subjects with exercise and carotid tests were submitted to angiography. As a consequence, there are few patients with a negative EE (24.4%) in the sample and prevalence of CAD could be higher in our group than in the global population. Ideally, all subjects scheduled for EE and carotid ultrasonography should undergo angiography. However, it seems unethical to submit to an invasive, ionizing radiation exposing and expensive procedure asymptomatic people after optimal lifestyle and pharmacological management without bad-prognosis EE. Other important limitation is that the coronary artery stenosis percentage was assessed visually and not by using more accurate tools such as intravascular ultrasound or optical coherence tomography or by physiological assessment of CAD stenosis in the cardiac catheterization laboratory (fractional flow reserve). This is a consequence of a retrospective study design, when some techniques were not available at the time of the angiography performance and it also reflects the usual clinical practice where intermediate stenosis are treated in case of a positive stress test and the methods mentioned before are used according to interventional cardiologist criteria, if negative or no stress test available. Comparison between carotid ultrasound and intracoronary imaging techniques in case of normal angiography could have helped to establish a better correlation between carotid and coronary artery disease, however the aim of the study was to find an association between carotid disease and significant and possibly flow limiting epicardial coronary stenosis causing chest pain. It is also important to keep in mind that this is a real life cohort study and using intravascular ultrasound or optical coherence tomography in people without intermediate CAD increases the cost and the duration of the procedure. Finally, there are 13.6% of patients with resting wall motion abnormalities, but we must consider that there are several conditions other than ischemic heart disease, such as cardiac sarcoidosis, myocarditis or cardiomyopathies that can also cause them.

## Conclusions

In conclusion, our study shows that carotid disease, in particular the presence of CP, is associated with significant CAD in patients submitted to EE. Its addition to EE does not improve sensitivity, specificity, predictive values, likelihood ratios, efficiency and AUC for significant CAD diagnosis; probably due to insufficient statistical power. However, CP reclassified one third of patients to very high-risk SCORE category despite a negative EE or CAD absence and these subjects benefit from aggressive primary prevention interventions.

## Abbreviations

AUC: Area under the curve
BP: Blood pressure
CAD: Coronary artery disease
CI: Confidence interval
CIMT: Carotid intima-media thickness
CP: Carotid plaque
DM: Diabetes mellitus
EE: Exercise echocardiography
ESC: European Society of Cardiology
FPG: Fasting plasma glucose
GFR: Glomerular filtration rate
NLR: Negative likelihood ratio
NPV: Negative predictive value
OR: Odds ratio
PLR: Positive likelihood ratio
PPV: Positive predictive value
PTP: Pre-test probability
ROC: Receiver Operating Characteristic
SCORE: European Systematic COronary Risk Evaluation.
